# Economic burden of dengue in urban Bangladesh: A societal perspective

**DOI:** 10.1371/journal.pntd.0011820

**Published:** 2023-12-05

**Authors:** Abdur Razzaque Sarker, Subrata Paul, Fatema Zohara, Zakir Hossain, Irfat Zabeen, S. M. Zahedul Islam Chowdhury, Maruf Ahmed, Nausad Ali, Raymond Oppong

**Affiliations:** 1 Population Studies Division, Bangladesh Institute of Development Studies (BIDS), Dhaka, Bangladesh; 2 Health Economic Unit, Institute of Applied Health Research, University of Birmingham, Birmingham, United Kingdom; 3 Health Economic Unit, Melbourne School of Population and Global Health, University of Melbourne, Melbourne, Australia; 4 Health Economics Unit, Health Services Division, Ministry of Health and Family Welfare, Dhaka, Bangladesh; 5 General Economics Division, Bangladesh Planning Commission, Dhaka, Bangladesh; Centers for Disease Control and Prevention, UNITED STATES

## Abstract

**Background:**

Dengue, a vector-borne disease, is a major public health problem in many tropical and subtropical countries including Bangladesh. The objective of this study is to estimate the societal cost of illness of dengue infections among the urban population in Dhaka, Bangladesh.

**Methods:**

A cost-of-illness study was conducted using a prevalence-based approach from a societal perspective. Costs attributable to dengue were estimated from a bottom-up strategy using the guideline proposed by the World Health Organization for estimating the economic burden of infectious diseases.

**Results:**

A total of 302 hospitalized confirmed dengue patients were enrolled in this study. The average cost to society for a person with a dengue episode was US$ 479.02. This amount was ranged between US$ 341.67 and US$ 567.12 for those patients who were treated at public and private hospitals, respectively. The households out-of-pocket cost contributed to a larger portion of the total costs of illness (66%) while the cost burden was significantly higher for the poorest households than the richest quintile.

**Conclusions:**

Dengue disease imposes a substantial financial burden on households and society. Therefore, decision-makers should consider the treatment cost of dengue infections, particularly among the poor in the population while balancing the benefits of introducing potentially effective dengue preventive programs in Bangladesh.

## Author summary

Dengue is a vector-borne viral disease affecting over 40 million people in Bangladesh. Dengue is endemic in urban Dhaka, Bangladesh, but the economic cost of illness is still limited. A growing literature shows that dengue imposes an enormous socio-economic burden on households, health systems, and societies. As there is no specific study estimating the economic costs of dengue infections in the Bangladesh context, the current study will serve as a benchmark to support evidence-based decision-making processes that guide the dengue prevention and control policies and allocation of health resources efficiently in Bangladesh. This study indicated, there is a substantial economic burden of dengue among the Dhaka city dwellers. We found that prevention of dengue hospitalization incidence, irrespective of the methods of prevention can save about US$ 479 per dengue case. Therefore, decision-makers should consider the treatment cost of dengue infections, particularly among the poor in the population while balancing the benefits of introducing potentially effective dengue preventive programs including vaccination strategies in Bangladesh.

## Introduction

Dengue, a vector-borne disease caused by the dengue virus, is a major public health problem in many tropical and subtropical countries [1]. The burden of dengue infections has dramatically increased over the past decades, with reports indicating that the number of symptomatic dengue infections doubled every ten years between 1990 and 2013 [2]. In recent years, outbreaks of dengue have occurred more frequently and extensively resulting in a significant proportion of severe cases and deaths in Bangladesh. In 2019, approximately 101,354 cases and 179 deaths were reported indicating the significant health and economic burden of dengue infections in Bangladesh [3]. In recent years, about 62,382 patients were hospitalized and 281 people died of dengue infections in 2022 [4] while most of the cases (about 84%) and deaths have been reported from urban Dhaka [5]. A risk factor study on the presence of dengue infections, conducted in urban Dhaka, observed that nearly 82% of the identified immature mosquitoes were *Aedes aegypti* which was responsible for dengue [6]. A recent study estimated that about 2.4 million people are infected with dengue yearly in Bangladesh, where most cases belong to major cities like Dhaka [7].

Dengue, a mosquito-borne viral disease, poses a significant threat to public health and imposes a substantial economic burden in Bangladesh. A growing literature shows that dengue imposes an enormous socio-economic burden on households, health systems, and societies [8,9]. A recent study observed that the annual economic burden of dengue infection was approximately US$950 million in Southeast Asia, while the average annual direct and indirect costs were US$451 million and US$499 million, respectively [10]. A study in India indicated that the annual economic cost of dengue was about US$ 5.71 billion in 2016 [8]. The global economic burden study found that the cost per episode is comparatively higher in low-income countries, approximately US$ 1,146, which was about 20 times higher than that of high-income countries (US$ 56) [11]. Although it is well assumed that dengue inflicts a high economic burden, there is hardly any data that can testify to this claim for Bangladesh. This information warrants that estimating the country-specific treatment cost of dengue is a must for a densely populated country like Bangladesh.

Estimating the costs of dengue disease for Bangladesh is also important because it helps policymakers and public health officials allocate resources and design interventions to control and prevent the disease. Additionally, cost estimates can be used to identify cost-effective interventions and to measure the economic impact of dengue control efforts over time. Hence, the overall objective of this study is to estimate the cost of illness of dengue infections among the urban population in Dhaka from a societal point of view. As there is no specific study estimating the economic costs of dengue infections in the Bangladesh context, the current study will serve as a benchmark to support evidence-based decision-making processes that will guide the dengue prevention and control policies and allocation of health resources efficiently in Bangladesh.

## Methods

### Ethics statement

This study has been approved by the ethical review board of the Bangladesh Institute of Development Studies (BIDS), under the protocol number PSD/EEBDB/07.10/20. This study was performed in line with the principles of the Declaration of Helsinki. Written informed consent was obtained from all respondents before data collection.

### Study settings and sample

This is a hospital-based cost-of-illness study conducted in two private and two public hospitals in urban Dhaka, Bangladesh. The hospitals were randomly selected based on a list of hospitals provided by the office of the Director General Health Service Division under the Ministry of Health and Family Welfare, Bangladesh. Public hospitals play a significant role in providing treatment for a relatively large population as the treatment cost is less than private for-profit hospitals. However, people frequently visit both public and private hospitals in Bangladesh, irrespective of socio-economic class. The hospitalized patients who suffered from dengue fever between January to December 2019 and were confirmed as ’dengue cases’ by a registered physician consist of our study subject. According to the central limit theorem, at least 30 positive cases should be required to estimate the mean cost with an assumption of normal distribution [12]. Thus, we randomly interviewed more than 30 dengue-confirmed male and female patients from each facility from the list of hospital patients. Moreover, a 10% non-response rate was assumed. However, after excluding all missing data, we finalized 302 participants as our study population. Respondents were the adult patients or the accompanying persons who were the most familiar with the costs incurred during the treatment of the patients. Patients’ records were also collected from the Hospital Records Departments (HRD). Self-reported expenses of dengue fever cases were then checked out and confirmed by patient registries and price data. Meanwhile, resource utilization data was also collected from the register books of a particular hospital. Informed consent was obtained from all respondents before the data collection process.

### Study perspective

The study was performed from the societal perspective, which means all types of costs were identified, measured, and valued no matter who incurred them. The societal perspective is the aggregation of the provider and household perspective recommended in the current standards for cost-effectiveness analysis methods [13].

### Cost estimates

Cost analysis was performed following the World Health Organization (WHO) guideline for estimating the economic burden of infectious disease [14]. A bottom-up micro-costing approach was used to generate the cost of illness per episode per patient where all relevant cost components were identified and valued at the most detailed level [15]. To capture the household cost of illness, both direct and indirect costs were captured. Direct costs are defined as expenditures during treatment by households consisting of direct medical costs and direct non-medical costs. Direct medical expenses include those costs that were borne for availing healthcare during dengue episodes, such as medicine, diagnosis, registration fees, and others. The direct non-medical cost includes the cost of transportation, lodging, food items, informal payment, and payment for helping the patients during the treatment procedure. The indirect cost includes the income loss as well as the cost productivity loss. These costs were borne due to visits to the health centers and absence from work and other activities related to dengue infection. Self-reported wage rates were used as a measure of income loss due to each episode. Human Capital Approach (HCA) was used to measure the productivity loss that reflected the value of all unpaid time devoted to caregiving themselves and family members, and friends [16].

We used age-specific and occupation-specific wage rates for capturing the productivity losses for non-market activities [17–20]. Following earlier studies, we used the age-specific wages for adults, teenagers, and children aged 5 to 14 years where the minimum salary rate according to the national level was attributed to the adult patients, one-half to the teenagers and three-quarters of that wage apply for children [21,22]. Half of the average salary rate was assigned to unpaid homeworkers considering their age group [17,18]. Intangible or psychic costs, such as costs related to suffering and grief, were excluded as those costs are not valued in the disease-specific cost of illness research [17]. The household cost burden was measured by the percentage of total household earnings devoted to dengue treatment [23].

The patient-specific treatment cost approach was taken to estimate the average treatment cost borne by the hospital according to WHO guidelines [14]. The costs include the diagnosis cost, laboratory cost, medicine cost, feeding cost, and other institutional costs borne by the hospitals for treating the dengue patient. Shared costs were allocated according to the number of patient’s days. Capital costs such as furniture, equipment was annuitized at a 3% discount rate as per WHO guidelines [15]. The provider’s actual cost of illness was calculated by the provider’s cost for treatment devoid of any fees received from the patients for hospitalization, drug, diagnostic tests, etc. In public healthcare facilities, the costs are often shared between the patients and hospitals. Indeed, in private hospitals, all treatment costs (including profit) are incurred by the households. However, as the Government initiated many special arrangements, we considered this aspect during the cost of illness estimation for dengue cases. Finally, the societal cost of illness was estimated by summing up providers’ actual cost of illness per patient with the cost incurred per household.

### Data analysis

Before the data analysis, missing answers and outliers were systematically verified. The patient-specific cost of illness borne by the households and provider costs were reported separately. Proportion, frequencies, rates, and ratio were presented with a standard deviation in the local currency, i.e., Bangladeshi Taka (BDT) and US dollars (US$) applying the exchange rate during the time of the survey (US$1 = 83.28 BDT during the end-point of the data collection year 2020). A sensitivity analysis was conducted to examine the effect of potential outlier on the total cost of illness to test the robustness of the assumptions [17]. One-way sensitivity analyses were presented in the tornado diagram. The data was analyzed using a spreadsheet in Microsoft Excel and Stata/SE 15.0 (StataCorp., College Station, TX, USA).

## Results

### Background characteristics of the study participants

A total of 302 hospitalized confirmed dengue patients were enrolled in this study (Table 1), of which approximately 54% (n = 163) was from public hospitals. The average age of the patient was 24 years and most of the patient were male (56%). Half of the patients (50%) came from a household with 3–4 members, followed by the larger households (35%). Around 87% of the total patients suffered from classic dengue fever while the other 13% of the patients suffered from Dengue Hemorrhagic Fever (DHF) or Dengue Shock Syndrome (DSS) and 4% of them required Intensive Care Unit (ICU) services. The average monthly household income was US$ 561.71 while the average monthly income for poorest households and richest households were US$ 35.35 and US$ 1668.60, respectively.

**Table 1:** Background information of the study participant, (n = 302).

Variables	Public hospitals (n = 163)	Private Hospitals (n = 139)	Overall (n = 302)
	n (%)/mean (SD)	n (%)/mean (SD)	n (%)/mean (SD)
**Age of dengue patient**			
Less than 6 years	22 (13.5)	17 (12.23)	39 (12.91)
6–18	52 (31.9)	32 (23.02)	84 (27.81)
19–50	78 (47.85)	77 (55.4)	155 (51.32)
50+	11 (6.75)	13 (9.35)	24 (7.95)
**Average age in years (mean, SD)**	22 (15.03)	26 (16.5)	24 (15.84)
Sex			
Female	76 (46.63)	58 (41.73)	134 (44.37)
Male	87 (53.37)	81 (58.27)	168 (55.63)
**Marital status**			
Married	59 (36.2)	63 (45.32)	122 (40.4)
Unmarried	78 (47.85)	59 (42.45)	137 (45.36)
Divorced/Widower/Widow	6 (3.68)	2 (1.44)	8 (2.65)
Not Applicable	20 (12.27)	15 (10.79)	35 (11.59)
**Education**			
No education	30 (18.4)	22 (15.83)	52 (17.22)
Up to primary	41 (25.15)	23 (16.55)	64 (21.19)
Secondary	53 (32.52)	27 (19.42)	80 (26.49)
Higher secondary	14 (8.59)	22 (15.83)	36 (11.92)
Higher	25 (15.34)	45 (32.37)	70 (23.18)
**Occupation of the dengue patient**			
Services	17 (10.43)	31 (22.3)	48 (15.89)
Business	6 (3.68)	15 (10.79)	21 (6.95)
Day labor	21 (12.88)	3 (2.16)	24 (7.95)
Students	58 (35.58)	40 (28.78)	98 (32.45)
Housewife	32 (19.63)	21 (15.11)	53 (17.55)
Retired	5 (3.07)	3 (2.16)	8 (2.65)
Not Applicable (aged under <5 yrs.)	22 (13.5)	15 (10.79)	37 (12.25)
Other	2 (1.23)	11 (7.91)	13 (4.3)
**Household size**			
Less than 3	24 (14.72)	21 (15.11)	45 (14.9)
3–4	93 (57.06)	59 (42.45)	152 (50.33)
5 and more	46 (28.22)	59 (42.45)	105 (34.77)
**Type of dengue fever**			
Classic dengue fever	145 (88.96)	119 (85.61)	264 (87.42)
DHF/DSS	18 (11.04)	20 (14.39)	38 (12.58)
**Length of Stay (LoS) in hospital (in days)**			
Less than 4	30 (18.4)	22 (15.83)	52 (17.22)
4–5	40 (24.54)	43 (30.94)	83 (27.48)
6–7	40 (24.54)	44 (31.65)	84 (27.81)
8–10	22 (13.5)	21 (15.11)	43 (14.24)
More than 10	31 (19.02)	9 (6.47)	40 (13.25)
**Average LoS in days (Mean, SD)**	7.00 (3.98)	6.19 (2.94)	6.63 (3.56)
**Admitted in ICU**			
No	157 (96.32)	133 (95.68)	290 (96.03)
Yes	6 (3.68)	6 (4.32)	12 (3.97)
**Average monthly income of the household (mean, SD)**	313 (199)	853.43 (1186.72)	561.71 (860.13)
**Household monthly income by income quintile (mean, SD)**			
Poorest	131 (35.66)	132 (39.40)	131 (36.35)
Poorer	247 (33.93)	254 (31.08)	249 (33.11)
Middle	371 (29.71)	367 (19.42)	369 (26.65)
Richer	546 (75.55)	594 (90.45)	575 (87.39)
Richest	1,111 (199.12)	1,756 (1730.61)	1705 (1668.60)

### The average cost per case: households perspective

The average cost for treating the dengue patient was US$ 406.06 (Table 2). The average total out-of-pocket (OOP) cost was US$ 316.51, representing 78% of the total household cost of illness. Direct-medical cost occupied approximately 55% of the total cost where the cost of diagnostic (US$ 20.17) and bed charges (US$ 17.22) were major cost drivers. Among direct non-medical costs, food cost (US$ 34.37) contributed 8.46% of the total cost of illness. However, the caregiver’s expenditure (US$ 22.91) was the critical cost component of direct non-medical costs, including food, mobile bill, transportation, and other related expenses borne by the caregivers during the treatment of dengue case. Along with direct cost, approximately 22% of the total cost of illness (US$ 89.55) was incurred due to patients’ and caregivers’ income loss. The household cost of illness of dengue patients becomes significantly high if the patient was treated in a private hospital (US$ 567.12) than being treated in a public hospital (US$ 268.72) as the public hospital is highly subsidized. The out-of-pocket costs were 81% and 72% of the total cost of illness for those treated at private and public hospitals, respectively. For private hospitals, the main cost drivers were bed fees (27%) and diagnostic fees (17%), followed by the cost of medicine (8%). However, the diagnostic cost (26%), food cost (12%), and the cost of medicine (9.5%) were the main cost driver for those who were treated in public hospitals in urban Bangladesh.

**Table 2:** Household cost of dengue treatment per-episode per-person, US$.

Type of cost	Cost components	Public hospitals (n = 163)					Private Hospitals (n = 139)			Overall (n = 302)		
		Mean	SD	% of total cost		Mean		SD	% of total cost	Mean	SD	% of total cost
Direct medical	Registration fee	0.18	0.31	0.07		4.72		1.98	0.83	2.27	2.64	0.56
	Consultation fee	-	-	-		26.62		42.24	4.69	12.25	31.54	3.02
	Diagnostic	68.62	84.68	25.54		97.47		119.55	17.19	81.90	103.05	20.17
	Medicine	25.61	35.67	9.53		44.97		44.76	7.93	34.52	41.20	8.5
	Plasma	13.68	59.15	5.09		23.97		105.75	4.23	18.41	83.89	4.53
	Medical equipment	2.73	7.28	1.02		4.91		4.24	0.87	3.73	6.16	0.92
	Bed charge	0.32	1.54	0.12		151.51		160.83	26.72	69.91	132.50	17.22
**Total direct medical**		**111.14**	**131.32**	**41.36**		**354.15**		**347.24**	**62.45**	**222.99**	**281.57**	**54.92**
Direct Non-Medical	Transportation	4.29	3.33	1.6		3.60		12.72	0.63	3.97	8.96	0.98
	Informal payment (e.g., tips)	2.32	3.90	0.86		3.55		5.94	0.63	2.89	4.98	0.71
	Food for patient	32.80	28.53	12.21		36.20		34.02	6.38	34.37	31.17	8.46
	Caregiver hiring	0.31	3.04	0.12		2.11		16.92	0.37	1.14	11.71	0.28
	Caregiver expenditure (e.g., food, mobile bill)	22.47	24.66	8.36		23.43		38.28	4.13	22.91	31.62	5.64
	Other cost (e.g., mug, mosquito coil)	4.31	6.80	1.6		25.68		27.92	4.53	14.15	22.27	3.48
**Total direct non-medical**		**66.50**	**49.51**	**24.75**		**94.60**		**92.41**	**16.68**	**79.43**	**73.69**	**19.56**
Costs incurred by other facilities	Cost incurred before the current hospital	11.76	18.35	4.37		9.32		17.84	1.64	10.64	18.12	2.62
	Cost incurred after discharge from the current hospital	5.03	16.83	1.87		1.61		7.90	0.28	3.46	13.57	0.85
**Total other facilities cost**		16.79	25.58	6.25	10.93			19.85	1.93	14.09	23.26	3.47
**Total direct cost**		**194.43**	**162.46**	**72.35**	**459.68**			**417.07**	**81.05**	**316.51**	**333.91**	**77.95**
Indirect cost	Patient’s indirect cost	28.78	42.63	10.71	55.19			166.25	9.73	40.93	117.57	10.08
	Caregiver’s indirect cost	45.51	43.32	16.94	52.26			92.17	9.21	48.62	70.11	11.97
**Total indirect cost**		**74.29**	**65.65**	**27.65**	**107.44**			**195.24**	**18.95**	**89.55**	**141.67**	**22.05**
**Total cost**		**268.72**	**201.90**	**100**	**567.12**			**533.81**	**100**	**406.06**	**418.06**	**100**

**Fig 1:**
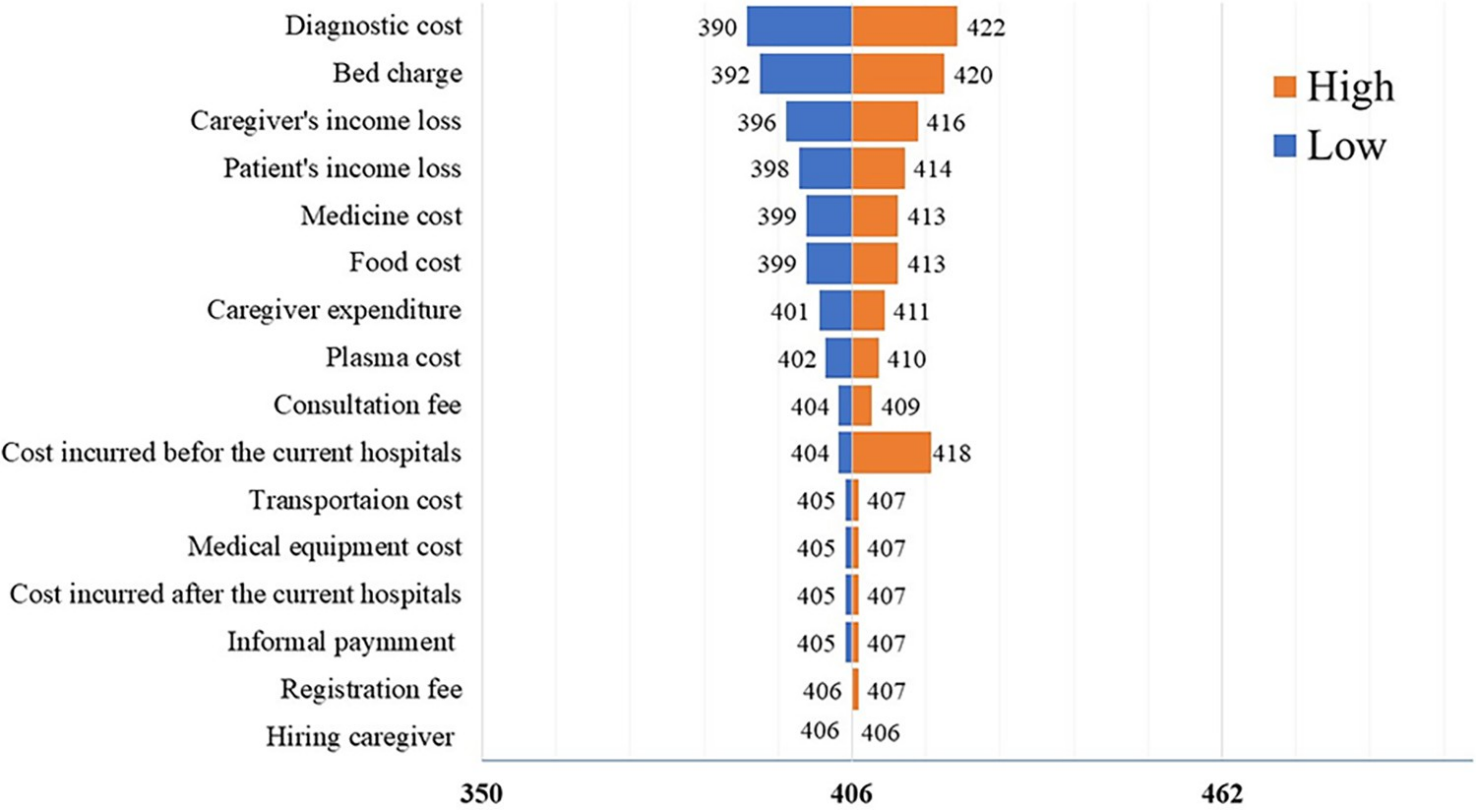
Tornado diagram.

We observed that the most influencing parameters were the cost of the diagnostic test, bed charge/fee, medicine, food cost and income loss of patients or caregivers during the treatment care (Fig 1).

### Household cost-burden and coping strategies

Table 3 shows the cost-burden due to dengue cases from households’ points of view. The OOP payment as a proportion of household income differed significantly among the income groups (P<0.001). The overall OOP expenditure due to dengue treatment was 56% out of monthly household income. However, in the poorest quintile, it exceeded 139% of the total household income while the richest (5th) quintile spent only 36% of monthly income of the households.

**Table 3:** Cost burden and catastrophic health expenditure in different socio-economic condition, US$.

Income group	Average monthly income (US$)	Average total OOP cost (US$)	OOP cost as a percentage of monthly household income
**Poorest quintile**	131.41	183.07	139.31%
**2nd quintile**	248.99	235.83	94.71%
**3rd quintile**	369.40	265.13	71.77%
**4th quintile**	574.65	338.65	58.93%
**Upper quintile**	1,704.61	616.61	36.17%
**Overall**	561.71	316.51	56.35%
***Rich-poor ratio***	0.16	0.04	*25*.*97%*
***Rich poor difference***	1,573.20	433.53	*-103*.*14%*

**Fig 2:**
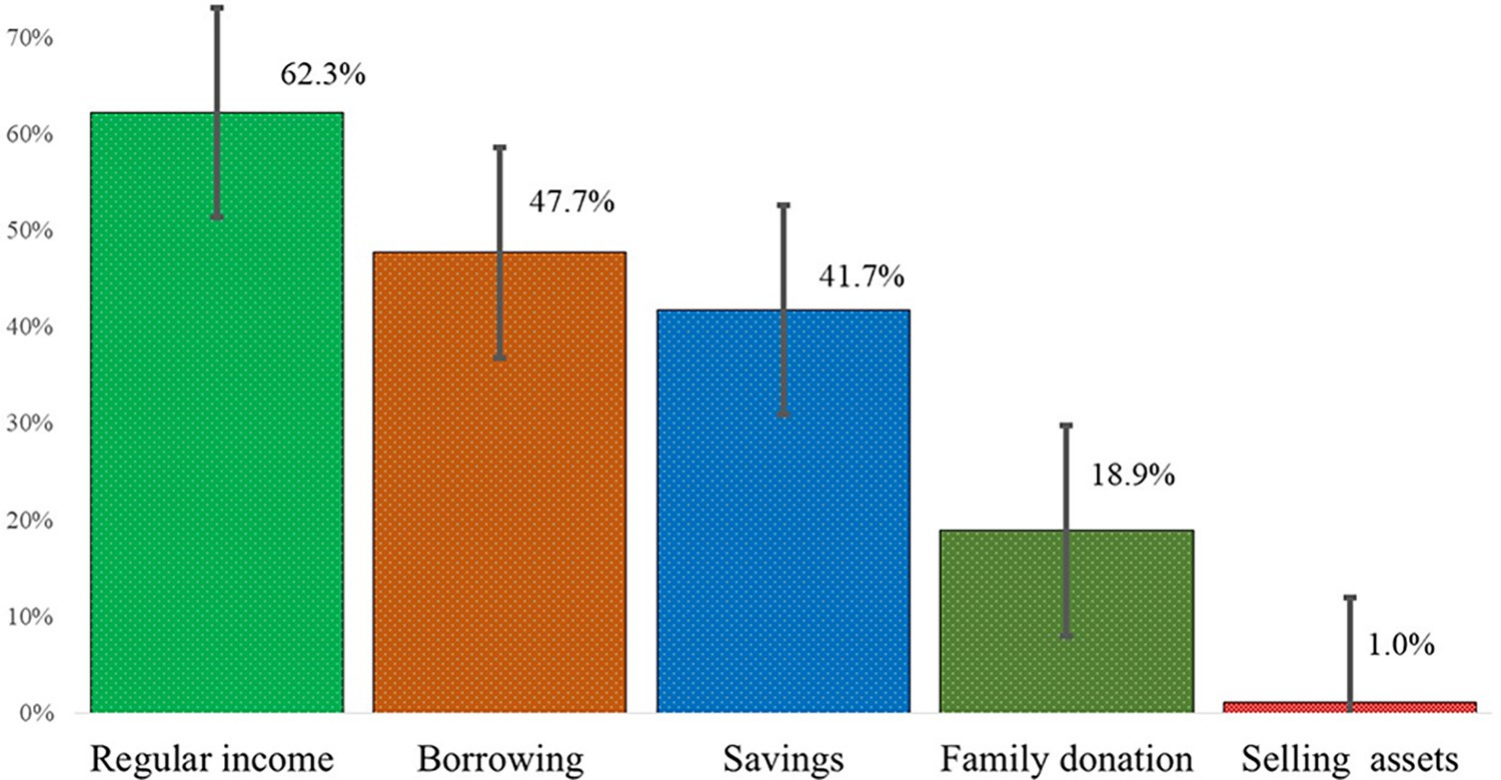
Coping strategies (multiple responses).

Fig 2 ***demonstrates*** the coping strategies during dengue treatment. The most common coping strategies were regular income (62.3%), borrowing from others (47.7%), savings (41.7%), a family donation (18.9%), and even selling permanent assets (1%).

### The average cost per patient: hospital perspective

Table 4 reports the estimated cost of dengue per case from public hospital perspective. Public hospitals are highly subsidized in Bangladesh; therefore, the total treatment cost is shared between households and public hospitals. The average total cost per episode per patient was US $73, while about 55% of the total cost (US$ 40.17) was used as direct medical costs such as medicine (3.53%) and medical staff (50.7%). Among the non-medical, the main cost drivers were shared personnel cost (16.5%), utilities (11.25%), and food cost for the patient (11%). A lump sum amount of capital costs such as cost of building (3%), furniture (1%), and recurrent cost such as cost of stationeries (1.33%) was also incurred during the treatment course of a dengue patient.

**Table 4:** Providers’ cost of illness per dengue patient: public hospital perspective (US$).

Type of cost	Cost component	Overall	
		Mean	% of total
**Direct Medical**	Diagnostic (e.g., CBC, NS1)	0.44	0.61
	Medicine	2.58	3.53
	Medical Materials (e.g., syringe, cannula)	0.12	0.17
	Staff personnel (e.g., doctor, nurse)	37.02	50.74
**Total direct medical**		40.17	55.05
**Direct non-medical**	Shared personnel (e.g., admin officer, accountant)	12.04	16.51
	Transport (e.g., ambulance)	0.14	0.19
	Food cost	8.06	11.05
	Stationeries (e.g., pen, print, paper, photocopy)	0.97	1.33
	Utilities (e.g., electricity, water, telephone)	8.21	11.25
	Laundry	0.43	0.6
	Capital items (e.g., furniture, machineries)	0.71	0.97
	Building (e.g., study ward/sq. feet)	2.22	3.05
**Total direct non-medical**		32.79	44.95
**Average cost per-patient (US$)**		**72.96**	**100**

### The average cost per patient: a societal perspective

Table 5 describes the average total cost of illness from a societal perspective. The average total societal cost of illness per dengue episode was US$ 479.02, whereas the average societal cost of illness amounted to US$ 342 and US$ 567 for those patients who were treated at public and private hospitals, respectively. Among all of the cost segments, household cost contributed a larger portion (85% of the total costs), and out-of-pocket contributed 66% of the total societal cost of illness, respectively. Considering the treatment cost borne by the provider, direct medical cost (8.4%) was the main cost driver, followed by non-medical costs (6.8%). It may be noted that in public hospitals, the total cost is shared between households and respective hospitals, which was not observed in private hospitals. The private hospital is a profit maximizer; therefore, the entire treatment cost was incurred by patients or their households. The indirect cost such as income and productivity loss of patients and caregivers consisted of 18% of the total societal cost of illness. We did not observe any significant differences in the cost of illness of dengue across the gender of the patient (see S1 Fig).

**Table 5:** Societal cost of illness due to dengue fever, per person per episode (US$).

Perspective	Type of cost	Public Hospital (n = 163)		Private Hospital (n = 139)		All patients (n = 302)	
		US $	% of total cost	US $	% of total cost	US $	% of total cost
**Household**	Direct medical	111	32.53	354	62.45	223	46.6
	Direct non-medical	66	19.46	95	16.68	79	16.6
	Cost incurred by other facilities	17	4.91	11	1.93	14	2.9
	Total Out-of-pocket cost	194	56.9	460	81.05	317	66.1
	Indirect (e.g., income loss)	74	21.74	107	18.95	90	18.7
**Total household**		**269**	78.65	**567**	100	**406**	84.8
**Provider**	Direct medical	40	11.75	-	-	40	8.4
	Direct non-medical	33	9.6	-	-	33	6.8
**Total provider**		73	21.35	-	-	73	15.2
**Societal**		**342**	100	**567**	100	**479**	100

## Discussion

Dengue is an emerging public health problem in Bangladesh. In recent years, outbreaks of dengue have grown to be more frequent, larger, and with a greater proportion of severe cases and deaths in Bangladesh. The economic aspect of dengue infections has not been studied adequately in Bangladesh. This study provides the first estimate for the economic cost of dengue disease in urban Bangladesh from the perspective of the society. Therefore, this study could be used to gain a better understanding of how dengue prevention would bring about health and economic benefits to urban society in Bangladesh.

This study found that the average societal cost of dengue was US$ 479, whereas the average societal cost of illness was US$ 342 and US$ 567 if the patients were treated in public and private facilities respectively in Bangladesh. The high share of out-of-pocket payments in the healthcare facilities demonstrates the economic hardship of the urban people in Bangladesh. A recent study conducted in a tertiary hospital in Bangladesh showed that the average cost of a hospitalized dengue patient was US$ 102 while 67% came from out-of-pocket cost [24]. However, that study was conducted in a single public medical college hospital which was highly subsided by the government and did not analyze the costs from the broader societal perspective. A recent cost of illness study indicated that the average direct cost was US$ 933.51 and US$ 720.39 for pediatric and adult dengue severe cases in India [9]. The current study indicated that the overall OOP expenditure was 56% of monthly household income which inflicts a catastrophic financial burden on urban households. However, in the poorest quintile, treatment cost was 139% of their household income, which means that the poorest households have to rely on other sources of expenditures like savings, borrowing from friends and families, and even selling assets. Therefore, dengue prevention can reduce the catastrophic financial burden experienced by many households in urban Bangladesh, particularly the lower-income households in urban Dhaka.

Various studies on the economic burden of dengue infection were conducted in different regions of the world, and the average costs of illness per episode vary by the settings of the studies. A recent study mentioned that the annual economic burden of dengue fever was almost U$ 1,384 million in Southeast Asia with a per capita cost up to $ 2.41 [25]. If we compare the average cost per dengue case, we observed that the average cost per dengue case in Bangladesh was lower than the average cost of dengue per episode in Thailand (US$ 573), Malaysia (US$ 947), Indonesia (US$ 791 to US$ 1250), Venezuela (US$ 627), Brazil (US$ 676), Panama (US$ 1,065) and higher than from India (US$ 432.2), Pakistan (US$ 358), Sri Lanka (US$ 32 to US$ 330) and in many North American countries like El Salvador (US$ 457), Guatemala (US$ 418) and Cambodia (US$115) [26–29]. Although our study observed that the total societal treatment cost of dengue was comparatively higher than that of the neighboring countries. This might be due to various reasons such as the differences in study settings (e.g., urban vs rural), sampling strategy (e.g., local vs national) and even for the seasonal variations [8,9]. For instance, a study conducted in Colombo, Sri Lanka observed that cost of hospitalization of an adult dengue patient was US$ 196 to US$ 866 in a dengue epidemic year [27]. A recent cost-estimation study in India reported that the costs per hospitalized non-fatal and fatal dengue illnesses were about US$ 325 and US$ 36,385 respectively [8]. Therefore, the results might be different varying on the contexts, which further urges that priority should be given to conduct a multi-country study in near future.

The findings highlighted the substantial economic burden of dengue among Dhaka city dwellers, which stresses on the urgent need for an effective national prevention strategy to perform considerable cost-savings besides reducing morbidity. Therefore, policy-makers should consider the treatment cost of dengue infections, particularly among the poor in the population while balancing the benefits of introducing potentially effective dengue preventive programs such as vaccine introduction. Indeed, vaccination is often considered to be the ‘best buy’ as it reduces the incidence of diseases and case fatalities, saves out-of-pocket expenditure, avoids productivity losses, mitigates the excessive pressure on the health system, and ensures equity [30]. While the economic evaluation of the dengue vaccination program has not been conducted yet, such studies might find that the dengue vaccination is the most cost-effective way for dengue in many settings [31,32]. Furthermore, by reducing dengue cases, many households can be protected from catastrophic expenditure (SDG target 3.8.2), and consequently, this action can contribute to achieving universal health coverage. At the same time, various preventive measures should be taken to prevent and control dengue, including eliminating mosquito breeding grounds; effective solid waste disposal; use of mosquito coils, aerosols, mosquito nets, cleaning of larval habitats like overhead tanks, groundwater storage tanks, air coolers, refrigerators and others. Dengue epidemics and outbreaks usually occur in the monsoon, and larval source destruction is the primary job for vector control. Therefore, the dengue control program should be initiated before the rainy season starts. Effective dengue control initiatives require a concerted effort among government agencies, city corporations, NGOs, and communities.

Several limitations might affect the interpretation of the outcomes. First, there may be recall bias and incorrect reporting primarily due to inaccurate memory during illnesses which could be under or over represent the real situation. Second, the inclusion of patients in a hospital setting indicates that they experienced relatively moderate to severe symptoms and, therefore, a proportion of mildly symptomatic patients may have remained undiagnosed and escaped inclusion. This study was limited among hospitalized dengue patients, but many episodes occurred at the community level which was not captured in this study. Thus, there is limited generalizability of using data from selected public and private health facilities informing the national policy and practices. Despite these limitations, our study has the most comprehensive list of costs for hospitalized dengue cases and identified the cost-driver during treatment in urban Dhaka to date. Further, the methods and analysis of results were presented in a straightforward manner so that anyone can compare similar study findings in other settings. Therefore, it is expected that the current study will be useful for informing the policymakers with the necessary knowledge for rational investment choice in preventing dengue infections from Bangladesh.

## Conclusion

There is a substantial economic burden of dengue among the Dhaka city dwellers. We found that prevention of dengue hospitalization incidence, irrespective of the methods of prevention can save US$ 479.02 per dengue case. Therefore, decision-makers should consider the treatment cost of dengue infections, particularly among the poor in the population while balancing the benefits of introducing potentially effective dengue preventive programs in Bangladesh. Further, this study allowed us to connect the economic impact of any public health interventions (either preventive or promotive) that can reduce the prevalence of dengue, which can be estimated from the data generated from this study. Further, dengue is a multifactorial disease requiring comprehensive approaches to address its spread effectively. Investing in sanitation can be a crucial aspect of reducing the vector breeding sites and the burden of dengue.

## Supporting information

S1 Fig Cost of illness (US$) across gender of the patient. (TIF)
