# Supplementary figures and images for: Economic burden of dengue in urban Bangladesh: A societal perspective
Source: PLoS Negl Trop Dis. 2023 Dec 5;17(12):e0011820. doi: 10.1371/journal.pntd.0011820 (PMC10723663; doi:10.1371/journal.pntd.0011820)

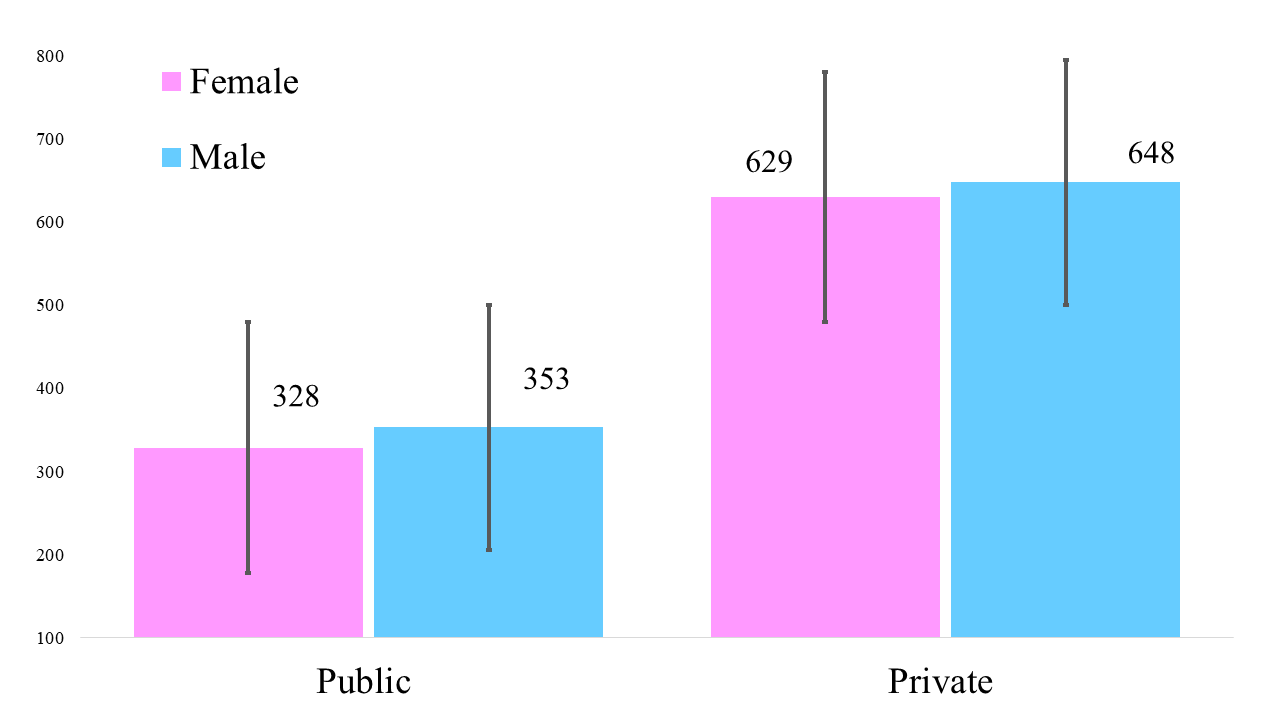

Supplement: S1 Fig — (TIF) [file pntd.0011820.s001.tif]
